# Health Tract, No. 44

**Published:** 1862-02

**Authors:** 


### HEALTH TRACT, No. 44.

### NEURALGIA,

From two Greek words, *Neuros*, nerve, and *Algos*, pain; means nerve-pain; but as there is no pain except in connection with the nerves, every pain or ache in the body is really "neuralgia." Ailments are generally named from the part affected, or the nature of the malady. "Head-ache," because the pain is in the head. "Pleuritis," or pleurisy, because there is inflammation, too much arterial blood in the *pleura*, or covering of the lungs. Neuralgia is always caused by bad blood; bad, because too poor or too much of it; too poor, because there is not exercise and pure air enough to secure a good digestion, and the person is thin and pale; too much blood, because there is too much eating, and the bowels not acting every day, more is taken into the system than passes from it, and it is too full. The person may be fleshy enough, and does not appear sick at all. For a week, live on cold bread and butter, fruits, and cold water. Take an enema of a pint or more of tepid water daily, and spend the whole of daylight in active exercise in the open air, and the neuralgia will be gone in three cases out of four—the feet being kept warm, and the whole body most perfectly clean. There are two kinds of neuralgia, sharp and dull; both caused by there being too much blood in or about the nerve. Perhaps arterial blood gives the sharp, venous blood the dull or heavy pain. In either case, the pain is of all forms of intensity, from simple discomfort to an agony almost unendurable. In the more fleshy parts, the pain is less severe, since the soft flesh yields before the distending nerve; distended by more and more blood getting into it, until it is occasionally three times its usual size; but when the nerve is in a tooth, or between two bones, or passes through a small hole in the bone, as in the face, or "facial neuralgia," which is neuralgia proper, or the *Tic Dolereux* of the French, the suffering is fearful, because there is no room for distension, and every instant, the heart, by its beating, plugs more blood into the invisible blood-vessels of the nerves. But in any such case, open a blood-vessel in the arm or elsewhere, until the person is on the very point of fainting, and the most excruciating neuralgia is gone in an instant, because the heart ceases to send on blood, and the blood already in a part, as naturally, flows out of it, as water naturally flows out of an uncorked bottle, on its side. Hence, a skin kept clean by judicious washings and frictions, helps, by its open pores, to unload the system of its surplus; the bowels kept free by fruits, berries, coarse bread, and cold water, is another source of deliverance of excess. While these articles of food supply but a moderate amount of nourishment, in addition, active exercise still more rapidly works off the surplusage of the system, and the man is well; not as soon as by the bleeding, but by a process more effective, more certain, more enduring, and without harm or danger. Hence, there is no form of mere neuralgia, which is not safely and permanently cured in a reasonable time by strict personal cleanliness, by cooling, loosening food, as named, and by breathing a pure air in resting in our chambers at night, and in moderate labor out of doors during the hours of daylight. Those who prefer uncertain physic or stimulants to these more natural remedies, are unwise, and ought to have neuralgia—a little. Half a dram (or half a tea-spoonful or thirty drops) of sal ammoniac, in one ounce (or two table-spoonfuls) of camphor-water. Dose: one tea-spoonful every five minutes until relieved, or from one to three tea-spoonfuls of valeriatae of ammonia thrice a day, are valuable temporary remedies.

